# EP06 COVID-19 masquerading as myositis and myopericarditis

**DOI:** 10.1093/rap/rkaa052.005

**Published:** 2020-11-03

**Authors:** Alexa José Escudero Siosi, Hudaifa Al Ani, Antoni Chan

**Affiliations:** Royal Berkshire Hospital, Reading, United Kingdom

## Abstract

**Case report - Introduction:**

Coronavirus (SARS-COV-19) typically targets the respiratory tract; however extra-respiratory manifestations such as myositis and myopericarditis may be the only presenting feature.

We present a patient with myopericarditis who developed sudden onset muscle weakness. CT thorax showed typical appearance of COVID-19 with an absence of respiratory symptoms. MRI of both thighs revealed diffuse symmetrical myositis. Her clinical and paraclinical abnormalities improved with the aid of steroids. We present our approach to the case and highlight that clinicians should consider myositis as another COVID-19 manifestation when reviewing the differentials.

**Case report - Case description:**

A 50-year-old female, non-smoker, presented with few days history of central chest pain radiating to her back. This was exacerbated by lying down and inspiration. Associated with mild shortness of breath on exertion. She denied upper respiratory tract symptoms.

Her past medical history included hypertension and myopericarditis in 2012 and 2013 requiring pericardiocentesis. In 2017 she presented with post-streptococcal erythema nodosum and reactive arthritis in left ankle.

On auscultation her heart sounds were normal, and chest was clear. Initial investigations revealed a mild lymphopenia 0.63, a C-reactive protein of 11mg/L, and a raised troponin 77 and 103 on repeat. D-dimer, Chest x-ray were normal. ECHO showed trivial anterior pericardial effusion, good biventricular function. Treatment included colchicine 500 micrograms four times a day and Ibuprofen 400 mg three times a day.

On her second day of admission she developed hypotensive episodes BP 75/49 mm/Hg and mild pyrexia of 37.5 degrees. Her chest pain continued. Electrocardiogram was normal, repeat echocardiogram showed stable 1.40 cm pericardial effusion. CT thorax revealed no dissection or features suggesting pulmonary sarcoidosis but ground-glass opacity changes in keeping with COVID-19. Her COVID-19 swab test came back positive.

On the 4th day of admission, she complained of sudden onset of severe pain affecting her thighs, shoulders, and arms, with marked proximal lower limbs and truncal weakness. Because of this, she struggled to mobilise. There was a rapid rise in her creatine kinase from 6.423U/L (day 5) to 32.230 U/L (day 7). ALT increased to 136. MRI showed diffuse myositis with symmetrical appearances involving the anterior, medial, and posterior muscle compartments of both thighs.

In view of her previous and current presentation, autoimmune screen and extended myositis immunoblot were sent and were negative. Interestingly, her clinical and paraclinical abnormalities improved dramatically after few days with no steroids initially.

**Case report - Discussion:**

The identification of extra-pulmonary manifestations neurological, cardiac, and muscular have recently increased as the number of COVID-19 cases grow.

This case highlights cardiac and skeletal muscle involvement could perhaps represent early or only manifestation of COVID-19.

Cardiac involvement in COVID-19 commonly manifests as acute cardiac injury (8–12%), arrhythmia (8.9–16.7%) and myocarditis. In our case the cardiac MRI demonstrated evidence of myocarditis in the basal inferoseptum and apex.

Myalgia and muscle weakness are among the symptoms described by patients affected by COVID-19. Some studies report the prevalence of myalgia to be between 11%-50%. The onset of symptoms and the fact that her symptoms improved rapidly led us to consider a viral myositis as the underlying cause, the viral component being COVID-19. We also considered other potential causes. There are reported cases of colchicine myopathy however this is more common in patients with renal impairment, which was absent in this case.

On further examination she did not have other clinical signs or symptoms of connective tissue disease or extra muscular manifestation of autoimmune myositis.

Her abnormal ALT may be derived from damaged muscle, and therefore in this context is not necessarily a specific indicator of liver disease. Interestingly abnormal liver function tests have been attributed in 16 - 53% of COVID-19 cases.

Little is known about the multiple biologic characteristics of COVID-19 and there are no established clinic serological criteria for COVID-19 related myositis nor useful values/cut offs to exclude cardiac involvement in myositis, further research is therefore warranted.

In conclusion, clinicians should be aware of the rare manifestation of COVID-19 and consider this in the differentials. Of course, it is important in the first instant to rule out any serious underlying disease or overlap disorder before attributing symptoms to COVID-19.

**Case report - Key learning points**
 

1. Myositis is a rare manifestation of COVID-19 that clinicians should be aware of.
2. Detailed medical history, examination and investigations identifies the most likely underlying cause.
3. In the right clinical context, COVID-19 – 19 testing should be included in baseline tests of patients presenting with myositis.

